# An efficient scheme for nonlinear shock wave model in a fractal domain under Caputo fractional operator

**DOI:** 10.1371/journal.pone.0302520

**Published:** 2024-06-10

**Authors:** Muhammad Nadeem, Yahya Alsayaad

**Affiliations:** 1 School of Mathematics and Statistics, Qujing Normal University, Qujing, China; 2 Department of Physics, Hodeidah University, Al-Hudaydah, Yemen; Universiti Tun Hussein Onn Malaysia, MALAYSIA

## Abstract

This paper introduces a refined approach for obtaining the analytical solution of the nonlinear shock wave model incorporating fractal derivatives. The Fractal Yang Variational Iteration Strategy (FYVIS) is utilized to obtain the approximate solution of a fractal model in the form of a series under Caputo fractional operator. The suggested method is the composition of the fractal Yang transform and the variational iteration approach. By using the two-scale fractal theory, we transform the fractal model into its traditional problem and then apply the yang transform to generate a recurrence relation. The variational iteration approach is now suitable to handle this recurrence relation without imposing any hypotheses or restrictions on variables. The derived results by the proposed scheme are shown in terms of series solution. Numerical calculations verify the accuracy and consistency of the suggested approach, demonstrating its excellent performance. The dynamic behavior of fractal components is explored by evaluating absolute errors and presenting two-dimensional diagrams across the fractal domain. This investigation underscores that the suggested technique offers an efficient and user-friendly solution for solving the nonlinear shock wave model involving fractal derivatives.

## 1 Introduction

In the seventeenth century, a myriad of mathematical scientists played pivotal roles in advancing fractal theory, contributing significantly to its modeling in natural phenomena. The exploration of fractal theory has garnered immense interest across diverse scientific and engineering disciplines, owing to its classical applications in various natural phenomena [1–3]. Fractional derivatives are frequently connected with fractal geometry, the relationships between fractional derivatives and fractal derivatives have yet to be resolved. Ivanovich [4] applied a finite difference scheme to numerically solve nonlinear fractal oscillatory systems with friction. Arqub and Maayah [5] used Fourier functions expansion rule to express the numeric analytic solutions by collection sets of orthonormal functions system. Carpinteri and Cornetti [6] delved into fractional calculus operators, specifically addressing stress and strain localization in fractal media. Elias-Zuniga et al. [7] ingeniously employed elliptic functions to derive approximate results for the Duffing oscillator in fractal form. Arqub [8] constructed an efficient computational reproducing kernel algorithm for the numerical solutions of singular Fredholm time-fractional partial integrodifferential equations subject to Dirichlet functions type. Lastly, He [9] contributed an efficient and straightforward method rooted in an ancient Chinese algorithm for solving nonlinear oscillators and determining their frequencies. These diverse approaches collectively highlight the multidimensional applicability and relevance of fractal theory in the scientific and engineering realms. The exploration of fractional calculus within the realm of partial differential equations holds significant interest across diverse scientific disciplines. Despite the challenges associated with obtaining solutions for these fractional models, particularly in the context of nonlinear problems lacking exact solutions, researchers have employed various strategies to address this complexity.

Recently, Dai and Yu [10] innovatively utilized the artificial neural network strategy to derive solutions for fractional differential problems, showcasing the efficacy of a modern computational approach. Dahmani and Anber [11] leveraged the variational iteration method (VIM) as a powerful tool for tackling nonlinear problems, demonstrating its applicability in the fractional domain. Alquran [12] introduced a residual power series scheme, providing an alternative approach to approximate the fractional foam drainage model with a focus on enhancing solution accuracy. Saifullah [13] proposed the modified double Laplace transform decomposition method for the analytical solution of time-fractional nonlinear Klein-Gordon equation and later Khan et al. [14] considered it for the solution of coupled Hirota and coupled Hirota Satsuma equations. Nadeem and Yao [15] presented the Laplace homotopy method, offering a systematic approach to derive solutions for fractional heat-like and wave-like problems with variable coefficients. Yasmin and Iqbal [16] delved into two-dimensional nonlinear systems, specifically third-order KdV equations and coupled Burgers equations, employing the Yang transform. Their study demonstrated the effectiveness of this scheme in yielding significant results towards exact solutions. Zhang et al. [17] employed the Yang-Laplace transform to analytically solve the fractal heat equation in the semi-infinite region, showcasing the versatility of transform methods.

The nonlinear shock wave equation, a partial differential equation lacking a general analytical solution, presents a nuanced extension of the classical shock wave equation. This evolved form incorporates fractional derivatives, elucidating the dynamics of nonlinear waves within various systems [18, 19]. He and Latifizadeh [20] combined the Laplace transform with VIM to numerically address the nonlinear partial differential equations, highlighting the synergy of different methodologies in obtaining accurate numerical results.

The pursuit of solutions to this intricate equation has spurred the efforts of numerous scientists and researchers, leading to the development of both analytical and numerical schemes for approximating solutions. Singh and Gupta [21] introduced the homotopy perturbation scheme to study shock wave problems with time-fractional order, presenting results in a series form. Allan and Khaled [22] employed the Adomian decomposition strategy, providing successive iterations for the shock wave problem and expressing results in a series context. Khatami [23] utilized the homotopy analysis method to calculate analytical results for the shock wave problem, specifically addressing its time derivatives. Further advancements in the approximate solution landscape for nonlinear fractional wave problems can be traced through various studies [24–26]. These endeavors collectively contribute to the ongoing exploration of robust methodologies, enhancing our ability to derive solutions for the complex nonlinear shock wave equation within diverse scientific and engineering contexts.

This paper introduces an innovative strategy for deriving the fractal solution of the nonlinear shock wave model featuring fractal derivatives. Termed the Fractal Yang Variational Iteration Strategy (FYVIS), our approach combines the fractal Yang transform and the variational iteration method. The methodological process unfolds in three distinct steps. Firstly, we leverage the two-scale fractal theory to transform the fractional model into its differential elements, laying the foundation for subsequent analysis. Following this, the fractal Yang transform is applied, facilitating the derivation of a recurrence relation without imposing any hypotheses or restrictions on variables. This transformative step enhances the adaptability of the method to a wide range of scenarios. In the final phase, the variational iteration approach is implemented to compute the recurrence relation, yielding solutions in the form of series solutions. One of the key advantages of this method lies in its ability to provide both exact and approximate analytical solutions for nonlinear models without imposing constraints on variables. Notably, the proposed scheme offers superior efficiency, requiring less processing than alternative techniques, while maintaining a high degree of numerical accuracy. The sections of this work are arranged as: Section (2) presents an overview of VIM, Section (3) outlines the methodology of the fractal Yang transform, and Section (1) explains the two-scale fractal theory. Section (5) presents the development of the FYVIS for solving fractal problems, and Section (6) demonstrates the application of the suggested technique to solve the nonlinear shock wave fractal model. We show some discussions on graphical illustration in Section (7), and provide a brief summary of findings in Section (8).

## 2 Variational iteration method

The Variational Iteration Method (VIM) serves as a semi-analytical tool for addressing nonlinear differential problems, originating from J.H. He’s extension of traditional perturbation methods in 1999. The fundamental concept behind VIM involves constructing a correctional functional that approximates the solution of a differential equation. Iteratively refining this functional leads to its convergence to the exact solution. The subsequent nonlinear partial differential equation is considered in the specified form [27]

$$\begin{array}{c} R\vartheta (\varsigma ,\eta )+N\vartheta (\varsigma ,\eta )=g(\varsigma ), \end{array}$$ (1)

where *R* is the linear operator, *N* is the nonlinear operator and g(ς,η) is a known analytical function. According to VIM, the correction functional is given by

$$\begin{array}{c} \vartheta_{n+1}\left(\varsigma ,\eta \right)=\vartheta_{n}\left(\varsigma ,\eta \right)+\int_{0}^{\varsigma }\lambda \left(\eta \right)\left[R\vartheta_{n}\left(\varsigma ,\eta \right)+N{\tilde{\vartheta }}_{n}\left(\varsigma ,\eta \right)-g\left(\varsigma \right)\right]d\eta , \end{array}$$ (2)

where λ is called Lagrange multiplier and can be determined optimally using the variational theory, ${\tilde{\vartheta }}_{n}$ is supposed to be a restricted component such that $\delta {\tilde{\vartheta }}_{n}=0$. The initial value of *ϑ*_0_ depends on the choice of the given conditions. Thus, the solution of (1) yields

$$\begin{array}{c} \vartheta =\underset{n\to \infty }{\text{lim}}\vartheta_{n}\left(\varsigma ,\eta \right). \end{array}$$ (3)

## 3 Idea of Yang transform

In this section, we provide the idea of Yang transform (YT) and its properties that are used to obtain the series results of a nonlinear shock wave model with fractal derivatives.

**Definition 3.1** The YT is defined as [28]

$$\begin{array}{c} Y\left[\vartheta \left(\eta \right)\right]=R\left(v\right)=\int_{0}^{\infty }e^{-\frac{\eta }{v}}\vartheta \left(\eta \right)d\eta , \end{array}$$ (4)

in which *R*(*v*) is YT of *ϑ*(*η*). Moreover, $\vartheta \left(\eta \right)=Y^{-1}\left[R\left(v\right)\right]$ is said to be inverse of YT.

**Perspectives**: The properties of YT in differential sense for a function *ϑ*(*η*) are expressed as [28]

$$\begin{array}{c} \begin{array}{cc} Y[\eta^{n}] & =n!v^{n+1},\\ Y[\vartheta^{\prime }\left(\eta \right)] & =\frac{R(v)}{v}-\vartheta \left(0\right),\\ Y[\vartheta^{\prime \prime }\left(\eta \right)] & =\frac{R(v)}{v^{2}}-\frac{\vartheta (0)}{v}-\vartheta^{\prime }\left(0\right). \end{array} \end{array}$$ (5)

In the same way, YT of *n*^*th*^ derivatives is defined as

$$\begin{array}{c} Y\left[\vartheta^{n}\left(\eta \right)\right]=\frac{R(v)}{v^{n}}-\sum_{k=0}^{n-1}\frac{\vartheta^{k}\left(0\right)}{v^{n-k-1}}. \end{array}$$ (6)

## 4 Two-scale fractal scheme

Water is continuous at all observing scales and the continuum mechanics holds whereas water turns to discontinuous at the molecule scale. The motion of a molecule in water cannot be described by continuum mechanics because it is chaotic, even if it is determined on the molecule scale. When an inappropriate scale is used, then uncertainty occurs. As a result, we require a new mathematical tool to deal with two-scale problems rather than the fractal dimension. The theory of two-scale is studied to convert the fractal space to a continuous one and defined as in *η* and *ς*-directions such that [29, 30]:

$$\begin{array}{c} \theta =\eta^{\alpha }, \end{array}$$ (7)

where *η* be a small scale and *θ* be the large scale, *α* be the fractal dimension. On a smaller scale, the nonlinear shock wave equation exhibits fluctuations, particularly at the highest point of the solitary wave. Conversely, a smooth solitary wave is predicted with a larger scale. Eq (7) provides an approximation for converting a small-scale fractal space to a large-scale smooth space. Consider the scenario of a tree that stops developing at night; when measured over 24 hours, it grows continuously, but when measured over 12 hours, its growth turns discontinuous. So, Eq (7) is also known as the two-scale fractal theory. The two-scale fractal theory makes it simple to convert fractal models for numerous problems into traditional models.

## 5 Idea of fractal Yang variational iteration method

This part describes the formulation of the FYVIS theory, which is utilized to generate the fractal results to the nonlinear shock wave fractal model. Let us assume the Eq (1) in the fractional form such as

$$\begin{array}{c} D_{\eta^{\alpha }}\vartheta \left(\varsigma ,\eta \right)=R\vartheta \left(\varsigma ,\eta \right)+N\vartheta \left(\varsigma ,\eta \right)-g\left(\varsigma \right), \end{array}$$ (8)

with initial condition

$$\begin{array}{c} \vartheta (\varsigma ,0)=h(\varsigma ), \end{array}$$ (9)

here *α* shows the fractal dimension.

**Step 1**: Now, according to the two-scale fractal theory [31], we obtain the Eq (8) such as

$$\begin{array}{c} D_{\theta }\vartheta \left(\varsigma ,\theta \right)=R\vartheta \left(\varsigma ,\theta \right)+N\vartheta \left(\varsigma ,\theta \right)-g\left(\varsigma \right). \end{array}$$

In other words, we can write as

$$\begin{array}{c} \frac{\partial \vartheta }{\partial \theta }=R\vartheta \left(\varsigma ,\theta \right)+N\vartheta \left(\varsigma ,\theta \right)-g\left(\varsigma \right). \end{array}$$ (10)

**Step 2**: By applying YT on Eq (10), we obtain

$$\begin{array}{c} Y[\frac{\partial \vartheta }{\partial \theta }]=Y[R\vartheta (\varsigma ,\theta )+N\vartheta (\varsigma ,\theta )-g(\varsigma )]. \end{array}$$

Thus, we get

Y[ϑ(ς,θ)]=vϑ(ς,0)+vY[Rϑ(ς,θ)+Nϑ(ς,θ)-g(ς)].

where *v* is transformation variable of Yang transform.

**Step 3**: The inverse of YT yields as,

$$\vartheta (\varsigma ,\theta )=h\left(\varsigma \right)+Y^{-1}[vY\{R\vartheta (\varsigma ,\theta )+N\vartheta (\varsigma ,\theta )-g(\varsigma )\}],$$ (11)

we also may write it as

$$\vartheta (\varsigma ,\theta )=p\left(\varsigma ,\theta \right)+Y^{-1}[vY\{R\vartheta (\varsigma ,\theta )+N\vartheta (\varsigma ,\theta )\}],$$ (12)

where $p\left(\varsigma ,\theta \right)=h\left(\varsigma \right)-Y^{-1}[vY\{g(\varsigma )\}]$.

**Step 4**: According to VIM, we get

$$\begin{array}{c} \vartheta_{n+1}\left(\varsigma ,\theta \right)=p\left(\varsigma ,\theta \right)-\int_{0}^{\varsigma }[\frac{\partial \vartheta_{n}\left(\varsigma ,\theta \right)}{\partial \theta }-\frac{\partial }{\partial \theta }Y^{-1}\{vY(R\vartheta (\varsigma ,\theta )+N\vartheta (\varsigma ,\theta ))\}]d\theta . \end{array}$$ (13)

Alternately,

$$\begin{array}{c} \vartheta_{n+1}\left(\varsigma ,\theta \right)=p\left(\varsigma ,\theta \right)+Y^{-1}\{vY(R\vartheta (\varsigma ,\theta )+N\vartheta (\varsigma ,\theta ))\}. \end{array}$$ (14)

We study the strength of this scheme for the upcoming mathematical applications.

**Step 5**: Now, converting this iteration series with the help of two-scale theory into fractal series. This fractal series yields the exact solution.

## 6 Numerical examples

In this part, we implement the idea of FYVIS for driving the fractal results of the nonlinear shock wave fractal model. The obtained iterations are very simple and converge to the exact solution very easily. Graphical visuals are demonstrated to observe the convergence of our proposed scheme. We use Mathematica program 11 for graphical and numerical results.

### 6.1 Example 1

Consider the nonlinear shock wave fractal model such as

$$\begin{array}{c} D_{\eta^{\alpha }}\vartheta +(\frac{1}{c_{0}}-\frac{\gamma +1}{2}\frac{\vartheta }{c_{0}^{2}})D_{\varsigma }\vartheta =0, \end{array}$$ (15)

with the initial condition

$$\begin{array}{c} \vartheta \left(\varsigma ,0\right)=e^{-\frac{\varsigma^{2}}{2}}, \end{array}$$ (16)

where *γ* is the specific heat, *c*_0_ and *γ* are constants. In [32], it is shown that if $c_{0}\gg \frac{1}{2}\left(\gamma +1\right)\vartheta$ then a series solution yield as

$$\begin{array}{c} \vartheta \left(\varsigma ,\eta \right)=\sum_{0}^{\infty }\frac{{\left(-\eta B\right)}^{n}}{(n+1)!}{\left(n+1\right)}^{n/2}H_{n}\left(\sqrt{n+1}\right)e^{-{\left(\varsigma -\eta /2\right)}^{2}\left(n+1\right)/2}, \end{array}$$

where $B=\left(\gamma +1\right)/2c_{0}^{2}$ and *H*_*n*_(.) is the Hermit polynomial of order *n*. The leading terms of the above-series solution will be expanded,

$$\vartheta (\varsigma ,\eta )=e^{-{\left(\varsigma -\eta /2\right)}^{2}/2}[1-\frac{5}{16}\eta \left(\varsigma -\eta /2\right)e^{-{\left(\varsigma -\eta /2\right)}^{2}/2}+\frac{25\eta^{2}}{512}[3({\left(\varsigma -\eta /2\right)}^{2}-1)]e^{-{\left(\varsigma -\eta /2\right)}^{2}}+\cdots ].$$ (17)

The two-scale fractal approach converts the Eq (15) such as

$$\begin{array}{c} D_{\theta }\vartheta +(\frac{1}{c_{0}}-\frac{\gamma +1}{2}\frac{\vartheta }{c_{0}^{2}})\frac{\partial \vartheta }{\partial \varsigma }=0. \end{array}$$ (18)

By applying YT on Eq (18), we obtain

$$Y[\vartheta (\varsigma ,\theta )]=vu\left(\varsigma ,0\right)-vY[\frac{1}{c_{0}}-\frac{\gamma +1}{2}\frac{\vartheta }{c_{0}^{2}}]\frac{\partial \vartheta }{\partial \varsigma }.$$

The inverse of YT yields as,

$$\vartheta (\varsigma ,\theta )=e^{-\frac{\varsigma^{2}}{2}}-Y^{-1}[vY\{\frac{1}{c_{0}}-\frac{\gamma +1}{2}\frac{\vartheta }{c_{0}^{2}}\}\frac{\partial \vartheta }{\partial \varsigma }].$$ (19)

According to Eq (13), we get

$$\begin{array}{c} \vartheta_{n+1}\left(\varsigma ,\theta \right)=\vartheta_{n}\left(\varsigma ,\theta \right)-\int_{0}^{\varsigma }[\frac{\partial \vartheta_{n}\left(\varsigma ,\theta \right)}{\partial \theta }+\frac{\partial }{\partial \theta }Y^{-1}\{vY(\frac{1}{c_{0}}\frac{\partial \vartheta_{n}}{\partial \varsigma }-\frac{\gamma +1}{2}\frac{\vartheta_{n}}{c_{0}^{2}}\frac{\partial \vartheta_{n}}{\partial \varsigma })\}]d\theta . \end{array}$$ (20)

Thus, we obtain the following iterations

$$\begin{array}{cc} \vartheta_{0}\left(\varsigma ,\theta \right) & =e^{-\frac{\varsigma^{2}}{2}},\\ \vartheta_{1}\left(\varsigma ,\theta \right) & =e^{-\frac{\varsigma^{2}}{2}}-[\frac{1}{c_{0}}-\frac{\gamma +1}{2c_{0}^{2}}e^{-\frac{\varsigma^{2}}{2}}]\varsigma e^{-\frac{\varsigma^{2}}{2}}\theta ,\\ \vartheta_{2}\left(\varsigma ,\theta \right) & =e^{-\frac{\varsigma^{2}}{2}}-[\frac{1}{c_{0}}-\frac{\gamma +1}{2c_{0}^{2}}e^{-\frac{\varsigma^{2}}{2}}]\varsigma e^{-\frac{\varsigma^{2}}{2}}\theta +e^{-\frac{\varsigma^{2}}{2}}[-\frac{1}{c_{0}^{2}}+\frac{\varsigma^{2}}{c_{0}^{2}}-\frac{\gamma +1}{c_{0}^{3}}e^{-\frac{\varsigma^{2}}{2}}\\ & -\frac{2(\gamma +1)}{c_{0}^{3}}\varsigma^{2}e^{-\frac{\varsigma^{2}}{2}}-\frac{{\left(\gamma +1\right)}^{2}}{4c_{0}^{4}}e^{-\varsigma^{2}}+\frac{3{\left(\gamma +1\right)}^{2}}{4c_{0}^{4}}\varsigma^{2}e^{-\varsigma^{2}}]\theta^{2},\\ & \vdots \end{array}$$

proceeding the same way, we can obtain the other components of *ϑ*_*n*_ and thus the iteration result can be arranged as

$$\begin{array}{cc} \vartheta (\varsigma ,\theta )= & e^{-\frac{\varsigma^{2}}{2}}-[\frac{1}{c_{0}}-\frac{\gamma +1}{2c_{0}^{2}}e^{-\frac{\varsigma^{2}}{2}}]\varsigma e^{-\frac{\varsigma^{2}}{2}}\theta +e^{-\frac{\varsigma^{2}}{2}}[-\frac{1}{c_{0}^{2}}+\frac{\varsigma^{2}}{c_{0}^{2}}-\frac{\gamma +1}{c_{0}^{3}}e^{-\frac{\varsigma^{2}}{2}}\\ & -\frac{2(\gamma +1)}{c_{0}^{3}}\varsigma^{2}e^{-\frac{\varsigma^{2}}{2}}-\frac{{\left(\gamma +1\right)}^{2}}{4c_{0}^{4}}e^{-\varsigma^{2}}+\frac{3{\left(\gamma +1\right)}^{2}}{4c_{0}^{4}}\varsigma^{2}e^{-\varsigma^{2}}]\theta^{2}+\cdots . \end{array}$$

Using the Eq (7), we get

$$\begin{array}{c} \begin{array}{cc} \vartheta (\varsigma ,\eta )= & e^{-\frac{\varsigma^{2}}{2}}-[\frac{1}{c_{0}}-\frac{\gamma +1}{2c_{0}^{2}}e^{-\frac{\varsigma^{2}}{2}}]\varsigma e^{-\frac{\varsigma^{2}}{2}}\eta^{\alpha }+e^{-\frac{\varsigma^{2}}{2}}[-\frac{1}{c_{0}^{2}}+\frac{\varsigma^{2}}{c_{0}^{2}}-\frac{\gamma +1}{c_{0}^{3}}e^{-\frac{\varsigma^{2}}{2}}\\ & -\frac{2(\gamma +1)}{c_{0}^{3}}\varsigma^{2}e^{-\frac{\varsigma^{2}}{2}}-\frac{{\left(\gamma +1\right)}^{2}}{4c_{0}^{4}}e^{-\varsigma^{2}}+\frac{3{\left(\gamma +1\right)}^{2}}{4c_{0}^{4}}\varsigma^{2}e^{-\varsigma^{2}}]\eta^{2\alpha }+\cdots . \end{array} \end{array}$$ (21)

Using Eq (3), this fractal series yields the exact solution as follows

$$\begin{array}{c} \vartheta =\underset{n\to \infty }{\text{lim}}\vartheta_{n}\left(\varsigma ,\eta \right), \end{array}$$

which is in full agrement with [24, 33].

### 6.2 Example 2

Consider another nonlinear shock wave fractal model such as

$$\begin{array}{c} D_{\eta^{\alpha }}\vartheta +\vartheta D_{\varsigma }\vartheta -D_{\eta^{\alpha }}\vartheta_{\varsigma \varsigma }=0, \end{array}$$ (22)

subjected to the condition

$$\begin{array}{c} \vartheta \left(\varsigma ,0\right)=3\;{\text{sech}}^{2}(\frac{\varsigma -15}{2}). \end{array}$$ (23)

The two-scale fractal approach converts the Eq (22) such as

$$\begin{array}{c} D_{\theta }\vartheta +\vartheta \frac{\partial \vartheta }{\partial \varsigma }-D_{\theta }(\frac{\partial^{2}\vartheta }{\partial \varsigma^{2}})=0. \end{array}$$ (24)

By applying YT on Eq (24), it yields

$$Y[\vartheta (\varsigma ,\theta )]=vu\left(\varsigma ,0\right)-vY[\vartheta \frac{\partial \vartheta }{\partial \varsigma }-\frac{\partial }{\partial \theta }(\frac{\partial^{2}\vartheta }{\partial \varsigma^{2}})].$$

The inverse of YT yields as,

$$\vartheta (\varsigma ,\theta )=3\;{\text{sech}}^{2}(\frac{\varsigma -15}{2})-Y^{-1}[vY\{\vartheta \frac{\partial \vartheta }{\partial \varsigma }-\frac{\partial }{\partial \theta }(\frac{\partial^{2}\vartheta }{\partial \varsigma^{2}})\}].$$ (25)

According to Eq (13), we get

$$\vartheta_{n+1}\left(\varsigma ,\theta \right)=\vartheta_{n}\left(\varsigma ,\theta \right)-\int_{0}^{\theta }[\frac{\partial \vartheta_{n}\left(\varsigma ,\theta \right)}{\partial \theta }+\frac{\partial }{\partial \theta }Y^{-1}\{vY(\vartheta_{n}\frac{\partial \vartheta_{n}}{\partial \varsigma }-\frac{\partial }{\partial \theta }(\frac{\partial^{2}\vartheta_{n}}{\partial \varsigma^{2}}))\}].$$ (26)

Thus, we obtain the following iterations

$$\begin{array}{cc} \vartheta_{0}\left(\varsigma ,\theta \right) & =3\;{\text{sech}}^{2}(\frac{\varsigma -15}{2}),\\ \vartheta_{1}\left(\varsigma ,\theta \right) & =3\;{\text{sech}}^{2}(\frac{\varsigma -15}{2})+9\;{\text{sech}}^{4}(\frac{\varsigma -15}{2})\;\text{tanh}(\frac{\varsigma -15}{2})\theta ,\\ \vartheta_{2}\left(\varsigma ,\theta \right) & =[-\frac{27}{2}\;{\text{sech}}^{6}(\frac{\varsigma -15}{2})+\frac{189}{2}\;{\text{sech}}^{6}(\frac{\varsigma -15}{2})\;{\text{tanh}}^{2}(\frac{\varsigma -15}{2})]\frac{\theta^{2}}{2}\\ & +[\frac{135}{2}\;{\text{sech}}^{4}(\frac{\varsigma -15}{2})\;{\text{tanh}}^{3}(\frac{\varsigma -15}{2})-\frac{63}{2}\;{\text{sech}}^{4}(\frac{\varsigma -15}{2}\;\text{tanh}\;(\frac{\varsigma -15}{2}))]\theta ,\\ & \vdots \end{array}$$

By following the same procedure, we may acquire the remaining elements of *ϑ*_*n*_ and then the iteration result can be arranged as

$$\begin{array}{cc} \vartheta (\varsigma ,\theta ) & =3\;{\text{sech}}^{2}(\frac{\varsigma -15}{2})+9\;{\text{sech}}^{4}(\frac{\varsigma -15}{2})\;\text{tanh}(\frac{\varsigma -15}{2})\theta \\ & -[\frac{27}{2}\;{\text{sech}}^{6}(\frac{\varsigma -15}{2})-\frac{189}{2}\;{\text{sech}}^{6}(\frac{\varsigma -15}{2})\;{\text{tanh}}^{2}(\frac{\varsigma -15}{2})]\frac{\theta^{2}}{2}\\ & +[\frac{135}{2}\;{\text{sech}}^{4}(\frac{\varsigma -15}{2})\;{\text{tanh}}^{3}(\frac{\varsigma -15}{2})-\frac{63}{2}\;{\text{sech}}^{4}(\frac{\varsigma -15}{2}\;\text{tanh}(\frac{\varsigma -15}{2}))]\theta +\cdots . \end{array}$$

Using the Eq (7), we get

$$\begin{array}{c} \begin{array}{cc} \vartheta (\varsigma ,\eta ) & =3\;{\text{sech}}^{2}(\frac{\varsigma -15}{2})+9\;{\text{sech}}^{4}(\frac{\varsigma -15}{2})\;\text{tanh}(\frac{\varsigma -15}{2})\eta^{\alpha }\\ & -[\frac{27}{2}\;{\text{sech}}^{6}(\frac{\varsigma -15}{2})-\frac{189}{2}\;{\text{sech}}^{6}(\frac{\varsigma -15}{2})\;{\text{tanh}}^{2}(\frac{\varsigma -15}{2})]\frac{\eta^{2\alpha }}{2}\\ & +[\frac{135}{2}\;{\text{sech}}^{4}(\frac{\varsigma -15}{2})\;{\text{tanh}}^{3}(\frac{\varsigma -15}{2})-\frac{63}{2}\;{\text{sech}}^{4}(\frac{\varsigma -15}{2}\;\text{tanh}(\frac{\varsigma -15}{2}))]\eta^{\alpha }+\cdots . \end{array} \end{array}$$ (27)

Using Eq (3), this fractal series yields the exact solution as follows

$$\begin{array}{c} \vartheta \left(\varsigma ,\eta \right)=3\;{\text{sech}}^{2}(\frac{\varsigma -15-\eta }{2}), \end{array}$$ (28)

showing agrement with [24, 33].

## 7 Findings and discussion

**Fig 1:**
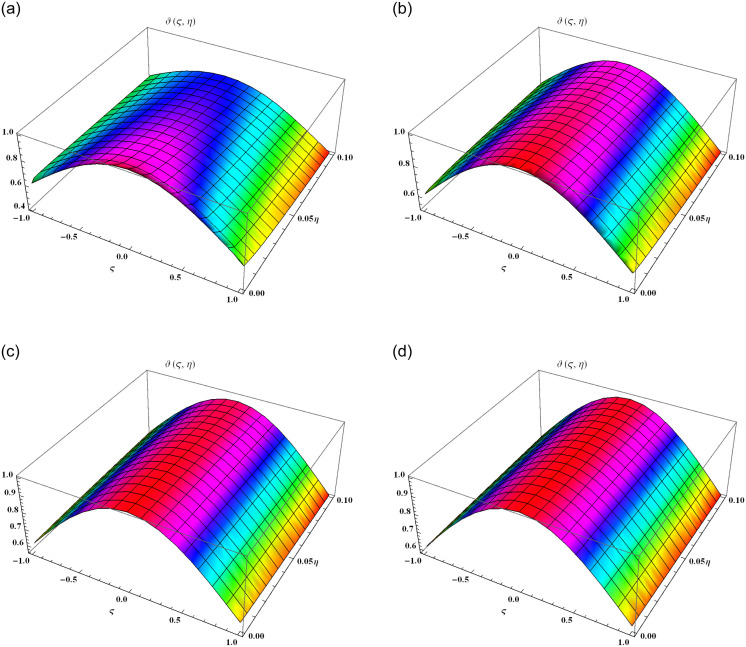
The surfaces solution of *ϑ*(*ς*, *η*) for distinct values of *α* within the domain −1 ≤ ς ≤ 1 and 0 ≤ *η* ≤ 0.1. (a) Graphical visual of *ϑ*(*ς*, *η*) at *α* = 0.25. (b) Graphical visual of *ϑ*(*ς*, *η*) at *α* = 0.50. (c) Graphical visual of *ϑ*(*ς*, *η*) at *α* = 0.75. (d) Graphical visual of *ϑ*(*ς*, *η*) at *α* = 1.

**Fig 2:**
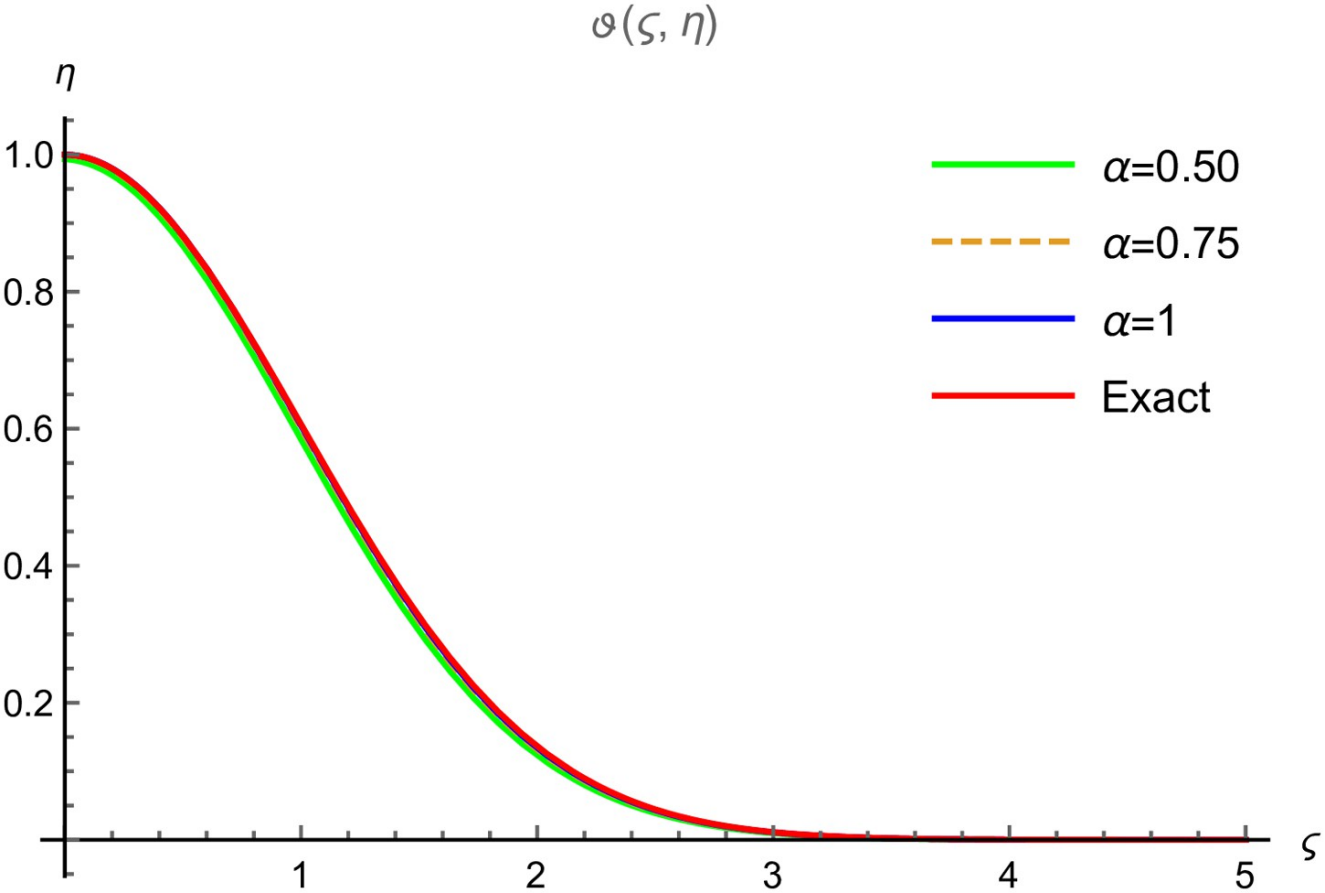
2D graphical comparison between exact and FYVIS results at different fractional order for Example (6.1).

The present section explains the graphical explanations of obtained results by using FYVIS. We observe that our proposed scheme handles the time fractional-order shock wave model perfectly and provides the fractal results very fast which leads to the exact solution. We consider the constant values with *γ* = 1.5 and *c*_0_ = 2 in our graphical and tabular representations. We have displayed the surface solutions of *ϑ*(*ς*, *η*) for various time fractional equations in Brownian. Fig (1a) display the surface solution for *α* = 0.25, Fig (1b) display the surface solution for *α* = 0.50, Fig (1c) display the surface solution for *α* = 0.75, Fig (1d) display the surface solution for *α* = 1. It is evident that *ϑ*(*ς*, *η*) decreases with the development of −1 ≤ *ς* ≤ 1 and 0 ≤ *η* ≤ 0.1 in Example 1. Fig 2 demonstrates the graphical error of *ϑ*(*ς*, *η*) at 0 ≤ *ς* ≤ 5 and 0 ≤ *η* ≤ 0.01 when *α* = 0.50 (solid green line), *α* = 0.75 (dotted line), *α* = 1 (solid blue line) and exact result (solid red line). In Table 1, we observe that the absolute error reduces its values when we have the minimum values of *ϑ* and *η*. To show the accuracy of the proposed scheme, we evaluate first few terms of the solution *ϑ*(*ς*, *η*) using Eq (17) obtained in [31] and compare these results with the solution of the proposed scheme using Eq (21).

**Table 1:** Absolute error among FYVIS and exact values across various fractional orders.

*ς*	*η*	FYVIS solution at *α* = 0.75	FYVIS solution at *α* = 1	Ref. [24]	Exact solution	Absolute error
0.5	0.001	0.881923	0.882397	0.882398	0.882393	4×10^−6^
	0.002	0.881512	0.882297	0.882298	0.882289	8×10^−6^
	0.003	0.881139	0.882195	0.882198	0.882185	0.00001
	0.004	0.870785	0.882092	0.882097	0.882081	0.000011
	0.005	0.880443	0.881989	0.881995	0.881978	0.000011
	0.006	0.880110	0.881884	0.881893	0.881874	0.00001
	0.007	0.879782	0.881778	0.881791	0.881770	8×10^−6^
	0.008	0.878459	0.881670	0.881688	0.881666	4×10^−6^
	0.009	0.879139	0.881562	0.881584	0.881562	000000
	0.01	0.878821	0.881453	0.881480	0.881459	6×10^−6^
1	0.001	0.05462	0.606342	0.606342	0.606527	0.000185
	0.002	0.604723	0.606153	0.606153	0.606523	0.00037
	0.003	0.604067	0.605963	0.605964	0.606519	0.000556
	0.004	0.603459	0.605773	0.605775	0.606515	0.000742
	0.005	0.602883	0.605582	0.605585	0.606510	0.000928
	0.006	0.602331	0.605390	0.605395	0.606505	0.001115
	0.007	0.601797	0.605198	0.605205	0.606500	0.001302
	0.008	0.601278	0.605005	0.605015	0.606494	0.001489
	0.009	0.600771	0.604812	0.604824	0.606489	0.001677
	0.01	0.600275	0.604617	0.604633	0.606482	0.001865

**Fig 3:**
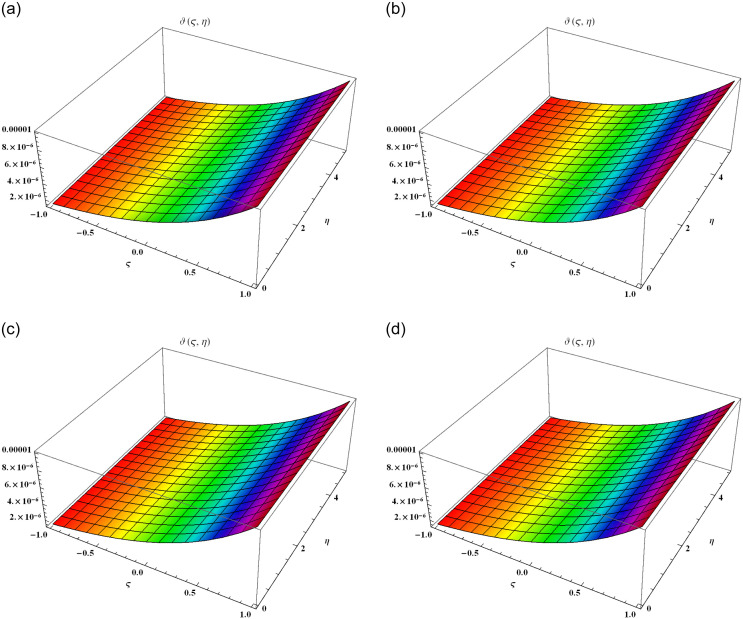
The surfaces solution of *ϑ*(*ς*, *η*) for different values of *α* within the domain −1 ≤ ς ≤ 1 and 0 ≤ *η* ≤ 5. (a) Graphical visual of *ϑ*(*ς*, *η*) at *α* = 0.25. (b) Graphical visual of *ϑ*(*ς*, *η*) at *α* = 0.50. (c) Graphical visual of *ϑ*(*ς*, *η*) at *α* = 0.75. (d) Graphical visual of *ϑ*(*ς*, *η*) at *α* = 1.

**Fig 4:**
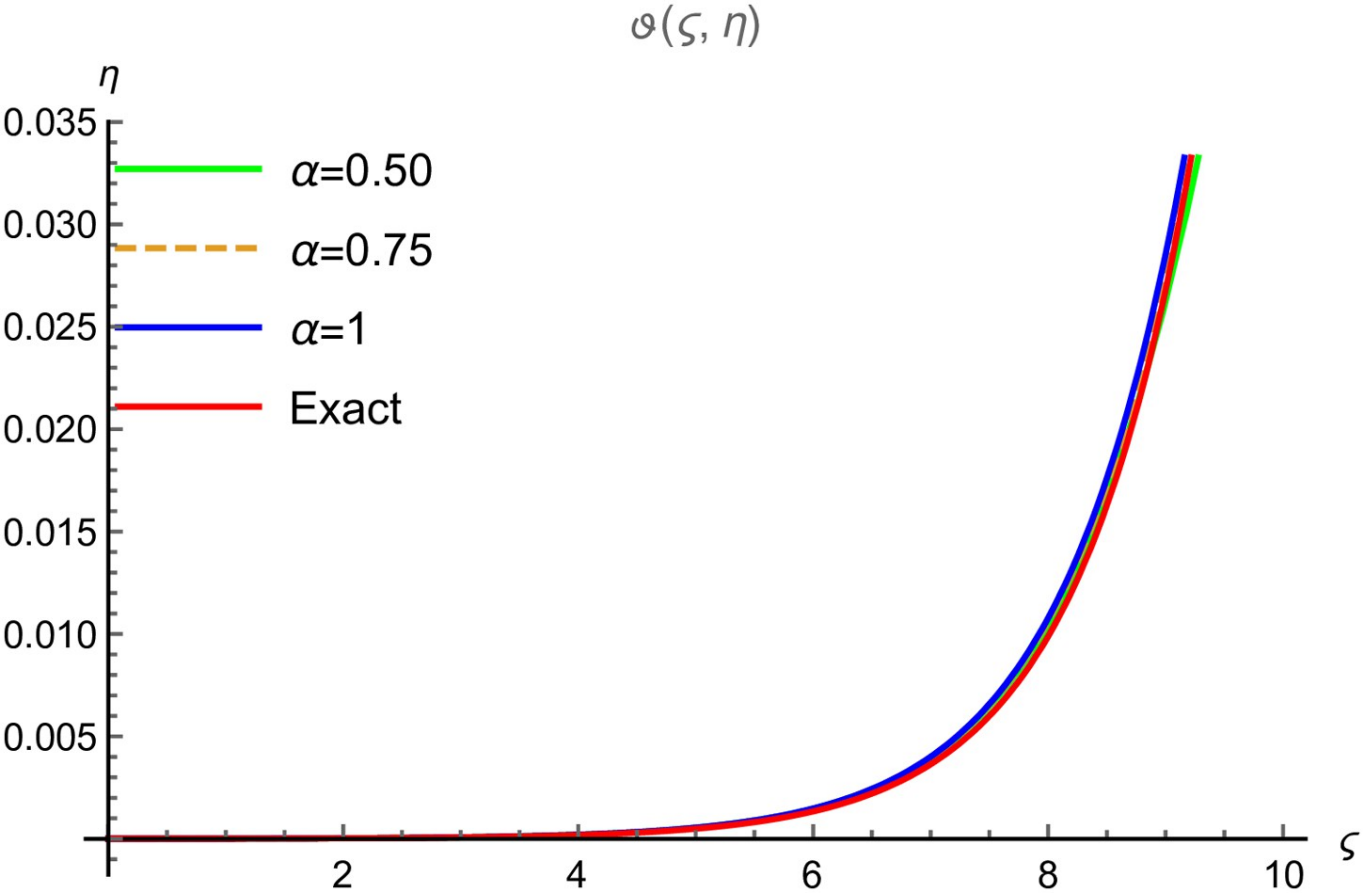
2D graphical comparison between exact and FYVIS results at different fractional order for Example (6.2).

We demonstrate the surface solution of *ϑ*(*ς*, *η*) for the fractal results derived by FYVIS and the exact solution in Example 2. Fig (3a) exhibits the surface solution for *α* = 0.25, Fig (3b) exhibits the surface solution for *α* = 0.50, Fig (3c) exhibits the surface solution for *α* = 0.75, Fig (3d) exhibits the surface solution for *α* = 1. The results of *ϑ*(*ς*, *η*) decreases with the increase of −1 ≤ *ς* ≤ 1 and 0 ≤ *η* ≤ 5. We show that the absolute error becomes minimum with the increase of *ϑ* and *η* in Table 2. Fig 4 demonstrates the graphical error of *ϑ*(*ς*, *η*) at 0≤ς≤10 and 0 ≤ *η* ≤ 0.1 when *α* = 0.50 (solid green line), *α* = 0.75 (dotted line), *α* = 1 (solid blue line) and exact result (solid red line). Only two iterations are used during numerical computation. It is obvious that by utilizing more terms, the accuracy of the results can be much improved, and the errors will converge to zero. By increasing the degree of *α*, the nonlinearity effects are affected despite reducing the wave amplitude. We observe that FYVIS is fully capable of handling the nonlinear shock wave fractal model.

**Table 2:** Absolute error among FYVIS and exact values across various fractional orders.

ς	*η*	FYVIS solution at *α* = 0.75	FYVIS solution at *α* = 1	Ref. [24]	Exact solution	Absolute error
2	0.01	0.0000271236	0.0000271237	0.0000271240	0.0000268539	2×10^−7^
	0.02	0.0000271234	0.0000271237	0.0000271241	0.0000265867	5.3×10^−7^
	0.03	0.0000271232	0.0000271236	0.0000271239	0.0000263222	8.0×10^−7^
	0.04	0.0000271230	0.0000271235	0.0000271237	0.0000260603	1.6×10^−6^
	0.05	0.0000271229	0.0000271234	0.0000271236	0.0000258010	1.3×10^−6^
	0.06	0.0000271228	0.0000271233	0.0000271235	0.0000255443	1.5×10^−6^
	0.07	0.0000271226	0.0000271232	0.0000271234	0.0000252901	1.8×10^−6^
	0.08	0.0000271225	0.0000271231	0.0000271233	0.0000250385	2.0×10^−6^
	0.09	0.0000271224	0.0000271230	0.0000271232	0.0000247893	2.3×10^−6^
	0.1	0.0000271223	0.0000271229	0.0000271231	0.0000245427	2.5×10^−6^
3	0.01	0.0000737276	0.0000737290	0.0000737295	0.0000729960	7.0×10^−7^
	0.02	0.0000737262	0.0000737283	0.0000737285	0.0000722697	1.4×10^−6^
	0.03	0.0000737249	0.0000737277	0.0000737279	0.0000715506	2.1×10^−6^
	0.04	0.0000737238	0.0000737270	0.0000737277	0.0000708387	2.8×10^−6^
	0.05	0.0000737227	0.0000737264	0.0000737268	0.0000701338	3.5×10^−6^
	0.06	0.0000737217	0.0000737257	0.0000737262	0.0000694360	4.2×10^−6^
	0.07	0.0000737208	0.0000737251	0.0000737256	0.0000687451	4.9×10^−6^
	0.08	0.0000737198	0.0000737244	0.0000737248	0.0000680611	5.6×10^−6^
	0.09	0.0000737189	0.0000737238	0.0000737242	0.0000673839	6.3×10^−6^
	0.1	0.0000737180	0.0000737231	0.0000737237	0.0000667134	7.0×10^−6^

## 8 Conclusion

In this research endeavor, we adeptly leverage the innovative Fractal Yang Variational Iteration Strategy (FYVIS) to successfully derive the fractal solution of a nonlinear shock wave model featuring fractal derivatives. A pivotal aspect of our approach lies in the incorporation of the two-scale fractal theory, a crucial element in constructing this scheme. Notably, our methodology distinguishes itself by eschewing the use of He’s polynomials and Adomian polynomials, sidestepping potential pitfalls that could compromise the fidelity of the actual problem.

One distinctive attribute of FYVIS is its independence from small values to identify the fractal solution, minimizing the introduction of unrealistic elements. This characteristic underscores the robustness and advantages of our suggested scheme. To assess the efficacy of our method, we meticulously evaluate the obtained fractal solution against the corresponding results of the exact solution, quantifying the accuracy through the computation of absolute errors. The juxtaposition of these errors, coupled with graphical representations, affirms that FYVIS is an exceptionally effective, dependable, and easy-to-implement series that converges swiftly to the exact solution.

Looking ahead, the proposed approach holds promise for extending its application to derive analytical approximate solutions for a diverse array of nonlinear fractal problems encountered in practical applications. Additionally, our future endeavors involve the integration of various fractional derivative operators with nonlinear fractal model systems, contributing to the ongoing exploration and advancement of methodologies in this dynamic field.
